# Repeated Insulin-Like Growth Factor 1 Treatment in a Patient with Rett Syndrome: A Single Case Study

**DOI:** 10.3389/fped.2014.00052

**Published:** 2014-06-03

**Authors:** Giorgio Pini, M. Flora Scusa, Alberto Benincasa, Ilaria Bottiglioni, Laura Congiu, Cyrus Vadhatpour, Anna Maria Romanelli, Ilaria Gemo, Chetti Puccetti, Rachel McNamara, Seán O’Leary, Aiden Corvin, Michael Gill, Daniela Tropea

**Affiliations:** ^1^Tuscany Rett Center, Versilia Hospital, Viareggio, Italy; ^2^Medical School, Trinity College Dublin, Dublin, Ireland; ^3^Department of Psychiatry, Trinity College Dublin, Dublin, Ireland

**Keywords:** Rett syndrome, insulin-like growth factor 1, social cognition, seizures, autonomic functions

## Abstract

Rett syndrome (RTT) is a devastating neurodevelopmental disorder that has no cure. Patients show regression of acquired skills, motor, and speech impairment, cardio-respiratory distress, microcephaly, and stereotyped hand movements. The majority of RTT patients display mutations in the gene that codes for the Methyl-CpG binding protein 2 (MeCP2), which is involved in the development of the central nervous system, especially synaptic and circuit maturation. Thus, agents that promote brain development and synaptic function are good candidates for ameliorating the symptoms of RTT. In particular, insulin-like growth factor 1 (IGF1) and its active peptide (1–3) IGF1 cross the Blood Brain Barrier, and therefore are ideal treatments for RTT Indeed, both (1–3) IGF1 and IGF1 treatment significantly ameliorates RTT symptoms in a mouse model of the disease In a previous study, we established that IGF1 is safe and well tolerated on Rett patients. In this open label clinical case study, we assess the safety and tolerability of IGF1 administration in two cycles of the treatment. Before and after each cycle, we monitored the clinical and blood parameters, autonomic function, and social and cognitive abilities, and we found that IGF1 was well tolerated each time and did not induce any side effect, nor it interfered with the other treatments that the patient was undergoing. We noticed a moderate improvement in the cognitive, social, and autonomic abilities of the patient after each cycle but the benefits were not retained between the two cycles, consistent with the pre-clinical observation that treatments for RTT should be administered through life. We find that repeated IGF1 treatment is safe and well tolerated in Rett patients but observed effects are not retained between cycles. These results have applications to other pathologies considering that IGF1 has been shown to be effective in other disorders of the autism spectrum.

## Introduction

The clinical case study performed in this experiment depicts the efficacy and safety of insulin-like growth factor 1 (IGF1) exposure upon two cycles of treatment. The patient is a young girl with a clinical and genetic diagnosis of Rett syndrome (RTT). The study started when the patient was 5 years old. The patient received first round of treatment on the 25th of May 2010 and discontinued on the 11th of November 2010. The second round of treatment started on the 20th of November 2012 and discontinued on the 28th of February 2013.

## Background

Rett syndrome is taxonomized as a pervasive neurodevelopmental disorder affecting mainly female children (1:10000) due to its X-linked method of inheritance (1). Methy-CpG binding protein 2 (MeCP2) dysfunction is associated with several neurodevelopmental disorders (1), and can cause a wide variety of phenotypes in females. Most males do not live past the first year due to neonatal encephalopathy and death in the classic RTT karyotype (2). The effects of MECP2 mutations lead to a loss of function or overexpression of the MeCP2 protein. If it results in a loss of function state, some examples of effects yield: classic RTT, atypical RTT, autism, and mild mental retardation (2). The majority of RTT patients (85%) have mutations in the MECP2 gene that lead to loss of MECP2 protein function. At this point of time, there is no cure to RTT syndrome, however treatment strategies are starting to emerge.

Rett syndrome is a progressive neurodevelopmental disorder that initially presents in females just past 18 months. Patients with RTT appear to have apparent normal development and milestone achievements up to the first 18 months of age (3). Early indication of neurological disease progression includes: deceleration of head growth, general growth retardation, weight loss, and weak posture via muscle hypotonia (2). Progression of RTT leads to the loss of purposeful use of their hands and the development of stereotypic hand wringing or washing movements: other hand movements are clapping, flapping, and mouthing (2). Withdrawal of social behaviors are noticeable where patients have shown symptoms such as irritability, self-abusive mannerisms, blank faces, audio hypersensitivity, reduction of eye contact, unresponsiveness to social cues, and indifference to variable environments (2). Eventual losses in motor coordination, ataxia, and gait apraxia are symptoms of motor pathway deterioration (2). Most girls eventually require wheel chair assistance during teenage years. Autonomic perturbations include hyperventilation (and other breathing abnormalities), seizures with variable severity, cardiac abnormalities, constipation, parkinsonian features, and oropharyngeal dysfunction (2). Patients may have a normal appetite but still experience weight loss and osteopenia. Structural abnormalities include scoliosis and rigidity. Behavioral complications involve teeth grinding, temper tantrums, depression, and anxiety. The condition eventually plateaus and the patient may live up to their seventh decade (2).

MeCP2 is an example of a mammalian protein that binds to methylated CpGs. For its ability to bind DNA and influence gene expression, MeCP2 is mostly believed to be a transcription factor (1), however localization of MeCP2 outside the nucleus and in the synaptic compartments (4) suggest that MeCP2 protein may have also functions directly related to synaptic activity. Inheritance of the MECP2 gene follows an X-linked dominant pattern and sporadic mutations are prevalent in 85% of classic RTT cases (2). Mutations that have been noted on the spectrum include missense, nonsense, and frame shift mutations for this protein. Mutations impacting on the nuclear localization sequence of MeCP2 or early truncating mutations have more severe phenotypes in comparison to missense mutations; while C-terminal deletions are thought to cause milder phenotypes. There is an association between the severity of the mutation and the severity of the phenotype (5). The phenotype of the female is highly variable depending on the expression levels of the unaffected copy of MECP2 on the other X chromosome.

The identification of the gene associated with Rett made possible the generation of mutant mice defective in MeCP2 functions. The mice have signs that resemble patients’ symptoms and therefore constitute a valid model for the study of neurobiology of Rett and for testing candidate therapeutics. A significant discovery has emerged from two independent groups concluding that in the mouse model, the reactivation of the natural Mecp2 gene in the adult mutant mice partially reestablished normal phenotype (6, 7). This finding implies that when the normal brain has impairment by a dysfunctional MeCP2, control conditions can be restored in the central nervous system. However, Zoghbi’s lab has shown that drug treatment for RTT has to be administered during the whole life of the patients (8).

Neurobiological analysis shows that MeCP2 mutations produce defects in central nervous system development, specifically in the maturation of circuits and synapses (4, 9). Therefore, medical treatments focused on brain development and synaptic functioning is promising for the regression of RTT. Indeed, a research study noted that overexpression of brain-derived neurotrophic growth factor (BDNF), which is a neurotrophin that has involvement in brain development and plasticity, resulted in the neutralization of the MeCP2 deficiency (10). This allowed for the consideration of growth factors, which are centralized around their involvement of neuronal and synaptic growth, toward potential treatment options for RTT syndrome. BDNF is incapable of crossing the blood brain barrier, so it is of no use for immediate pharmacological therapy consideration. However, IGF1 and its active peptide (1–3) IGF1, are capable of crossing the blood brain barrier. This quality makes them an ideal drug for treatment in neurodevelopmental disorders like RTT. Research has shown that IGF1 peptide treatment partially restored spine density and synaptic amplitude in the mouse model (9). The treatment was started at 2 weeks of age and was noted to stabilize cortical plasticity to the wild-type level. One of the major effects noted in this study was after the treatment of IGF1; MeCP2 KO with treatment mice improved from an average of 60-day lifespan to an average of 90-day lifespan in comparison with KO without treatment mice. Not only did the KO mice with treatment have increased life span, but also improved respiratory, cardiac, and locomotor function. In the study from Tropea et al. (9) (1–3) IGF1, has already shown to ameliorate the symptoms of RTT in the mouse model of the disease. IGF1 has also been shown to be an effective therapy in MeCP2 KO mice (11). Other studies show that IGF1 is a suitable treatment for human RTT patients and that there are no known risks associated with IGF1 administration (12, 13). In our previous study, six girls with classic RTT, between 4 and 11 years of age, were administered IGF1 subcutaneously twice per day for 6 months. They were evaluated based on the effectiveness of the treatment and it was reported that IGF1 partially restored a significant amount of the symptoms of RTT. From this study, all families and caretakers noticed an improvement in cognitive abilities of the girls and their interactions with the surrounding environment. Considered McGraw’s study, life-long therapy is necessary to combat RTT syndrome, like IGF1 for MeCP2 dysfunction (8).

The aim of this paper is to evaluate if repeated cycles of IGF1 is a suitable method, in respect to safety and efficacy, for a life-long treatment option in the human model. The clinical case study in this report depicts a patient receiving two cycles of IGF1 treatment and clinical parameter assessments. Also, we provide insight on how the patient responds after the treatment with two clinical evaluations after the treatment cycles. The study is an open label study, and the child displays classic RTT syndrome, where the phenotypic onset of the disease took place between the eighth and ninth month. The child started the first IGF1 cycle in May 2010, when the child was in the fifth year of age. IGF1 was administered twice daily (0.1 mg/kg) for 6 months. Two years later, another cycle of treatment was started. The dosage and routes of administration were the same, but the second cycle lasted 4 months. In between each cycle, blood parameters were monitored and autonomic responses evaluated. During both cycles, IGF1 was well tolerated in each cycle and no direct side effect was observed. IGF1 is also shown to cause no interference with the anti-epileptic medications that the patient was undergoing. Based on the conclusion of this experiment, the treatment is believed to promise a strong impact on RTT syndrome.

## Subjects and Methods

### Clinical analysis

We evaluated the RTT patient’s overall physical health, RTT-associated phenotypic expressions of the patient, and growth developments. We took notes, recorded in the Section “Results,” during various stages of the treatment process during two cycles of IGF1 exposure. The parents kept a diary with daily annotations of glycemia levels and alterations in behavior (sleep, eating, etc.)

#### Patient screening

Patient was chosen based on the presentation of classic RTT syndrome and confirmed mutation in the MECP2 gene. The family gave written consent for partaking in this study before any treatment took place. The selection criteria are described in Pini et al. (12).

#### Treatment administration

Mecasermin–Increlex (IGF1) was subcutaneously administered twice daily (0.1 mg/kg) in two cycles: the first cycle lasted nearly 6 months, and the second cycle lasted 4 months, with a 2 years gap between the two cycles. Possible side effects include hypoglycemia (headache, dizziness, nausea, and perspiration), tonsil hypertrophia, and local irritation at the injection site. The family was thoroughly informed about the treatment process; the caretakers of the patient were trained to perform daily injections, and were provided with a kit to measure glycemic levels. Patients were observed for side effects during every visit and critical events were recorded and are in summarized in the Section “Results.”

#### Post treatment examination

The patient was followed up twice after each cycle to evaluate general health after IGF1 exposure. Patient was physically examined based on cardio-respiratory factors, body growth parameters, and motor abilities. Critical events were recorded along with the corresponding epileptic treatment at that time. Behavioral examination along the parameters of social interaction and mood were also evaluated.

### Video analysis of cognitive and social abilities

Video analysis utilized approximately 20 h of video recordings of RTT patients and was filmed in Versilia Hospital (Italy). The RTT patient observed in this paper was an analyzed participant in this study. The investigators, analyzing the videos, were not aware that the patient had received IGF1 treatment. Observed preliminary footage from additional patients yielded the design of the scoring criteria. It was decided to score the girls on 20 features, 10 negatives, and 10 positives. The patient was scored in each feature on a scale from 1 to 5, with 1 indicating extreme RTT, 2 indicating severe RTT, 3 indicating moderate RTT, 4 indicating mild RTT, and 5 indicating absent RTT. In this way, a lower score was related to a more prevalent RTT-like behavior. The 10 negative features chosen were: hand wringing, biting, rocking, hitting, indiscriminate moaning, tongue chewing, vacant staring, bruxism, breath-holding/apnea, and Valsalva maneuver. The 10 positive features chosen were: pointing, manipulating, reaching for something, ability to mimic/imitate, indicating yes/no with head gesture, reactivity to a call, reactivity to an object, smiling in response to a stimulus, deliberate vocalization, and attention.

## Results

The patient was born from the mother’s first pregnancy. The mother suffers from epilepsy and was treated with anti-epileptic drugs (AEDs) since adolescence. The patient was born at week 38 of gestation by cesarean section due to an emergency response. At birth, the patient had the following dimensions: 2600 g in weight, 45.5 cm in length, and 33 cm in head circumference. The newborn received artificial feeding due to maternal agalactia. At the approximate age of 9 months, the child presented with: frequent pyloric stenosis, decreased stimulus to environment, mentally absent, and showed stereotypic hand to mouth and washing movements. The patient’s motor development showed independent seat positioning acquired at 8 months with kyphosis of the bust: unimanual walking with support at 18 months with mild postural tremor. At 18 months, the patient’s use of hands was discrete and she could grab objects and press buttons. The patient’s language development showed production of vocalization and infrequent simple syllabic doublings (bi-bi-, ma-ma, ba-ba). The patient showed bruxism with variable power. The patient had tendency to be constipated and sphincter control was not acquired. The sleep–wake rhythm was characterized by frequent nocturnal awakening and was prescribed Niaprazine (Nopron). On the 2nd of August 2006, genetic testing for RTT indicated a mutation c. 397C > T (R133C) of exon 4 of the MECP2 gene.

The clinical case study performed in this experiment depicts the efficacy and safety of IGF1 exposure upon two cycles of treatment. Careful consideration was executed in monitoring to the effects of IGF1 in both autonomic function and blood parameters. The patient received first round of treatment on the 25th of May 2010 and discontinued on the 11th of November 2010. The second round of treatment started on the 20th of November 2012 and discontinued on the 28th of February 2013. The dosage for IGF1 was the same in both cycles: 0.1 mg/kg maintenance dosages and 0.05 mg/kg first and last weeks were adjustment dosages. Our findings are noted below during each visit with the patient during their cycles of IGF1 treatment.

### First cycle of IGF1 treatment

The first experimentation with IGF1 started on 25th of May 2010 (first cycle), when the patients were 5 years and 8 months. We clinically assessed the patient and results were noted as follows: patient has slight ataxic ambulation, which required minimum support; slight hypotonia was noticeable and babinsky positive ROT hyper-excitability; wide-spread bouts of tremor often associated with tachypnea, and possible grasping of objects upon request. Patient demonstrated expressive language, characterized by varied and modulated vocalizations. The patient had a calm demeanor with discreet interest in external environment and surrounding people.

Second visit was on the 31st of August 2010. The patient showed good clinical health and similar ambulation as the first visit. The patient showed coastal navigation with the family. The hypotonia and wide-spread tremor was the same as first visit. The patient showed improvement in grip of objects. The patient continued to demonstrate expressive language, characterized by varied and modulated vocalizations. Communicative intentions were observed and actions were occasionally preceded by anticipatory behaviors. The demeanor remained calm.

Third visit took place on the 23th of November 2010. The patient maintained good general clinical condition and the parents reported a good clinical outcome at home. The patient was able to ambulate with unimanual support with tendency to externally rotate the march of the lower limbs, and launch of the same. The use of hands to engage in patterns of action with objects have been a new observation, such as pressing a button or lever to get a visual or sound effect. The patient demonstrates good conduct and interaction with unfamiliar people. The food and sleep–wake rhythm is regular, while obstinate constipation persists.

The patient was discontinued from IGF1 on the 27th of November 2010 and clinical assessments were continued.

The fourth visit was on the 15th of February 2011. The clinical observations were almost unchanged from the previous visit. The gait seemed to have improved, even when the child fatigued easily. There was a submission of critical events that took place. On the 8th of February 2011, at 10:40 a.m. the first epileptic crisis lasting 2–3 min was characterized by tremor, hypotonia, eyes wide and staring into space. This was followed by sleep. Secondary crisis showed facial and perioral cyanosis, tremor, and blank stares, followed by sleep. A third crisis at 6:10 p.m. noted guttural verses, staring, stiffening, and blank stares followed by sleep. A fourth crisis occurred at 7:30 p.m. where stiffening, staring, perioral cyanosis, pallor of the face, and strange vocalizations followed by sleep. Vomiting, facial and perioral cyanosis, and guttural sounds characterized the fifth crisis at 9:30 p.m. On the 9th of February 2011, the patient had a fever of 37.3°C. At 12:35 p.m., the first crisis lasted a minute and was characterized by salivation, perioral and facial cyanosis, absent eyes, and potential hypotonia causing the patient to fold on itself.

The second crisis (on February 9th) occurred at 1:38 p.m. and lasted 3 min; characterized by perioral and facial cyanosis and mouth tremors followed by sleep. The third crisis occurred at 5:27 p.m. and lasted for 2 min, characterized by an absent gaze. On the 11th of February 2011 at 10:16 a.m., the patient was noticed to have staring eyes and making guttural sob like sounds. On the 12th of February 2011 at 10:00 a.m., the patient woke up with a frightened look, began trembling with tears, and guttural vocalizations. A second crisis occurred at 3:40 p.m. characterized by neck muscle failure and appearance of sleep while sitting on the toilet, with guttural vocalizations. On the 13th of February 2011, the patient presented with a fever of 38.3°C, pharyngitis, and began Zithromax treatment.

The fifth visit took place on the 24th of May 2011. The clinical examination showed good general condition and parents reported a stable clinical experience. The child had unimanual-supported ambulation with tendency to externally rotate and launch of the lower limbs. The clinician noted a decreased resistance during walking. The patient was observed to have a discreet use of hands when she grabbed objects and pressed buttons to get a visual or sound effect. Social interaction showed observation of the environment and the maintenance of eye contact for a longer duration of time. The patient maintained a serene mood. The food and sleep–wake rhythm were regular and constipation still persisted. Critical incidents occurred almost daily and were characterized by staring, gaze deviation to the left, and clonus of the limbs. On the 13th of April 2011, the child presented a crisis with the semiotics for a 10-min period, which required Micronoan administration. The patient’s anti-epileptic therapy dosage was increased: valproate 200 + 200 mg/day.

The sixth visit took place on the 21st of October 2011. The clinical examination reported a good general clinical condition. The parents informed that the child was attentive, observational of the environment, interacting with people, and mood was serene. The critical events were reported to occur on awakening and were characterized by wide-eyed, clonus of the limbs, apnea, and cyanosis of the face. The patient then took Valproate: dosage of 250 + 200 mg/day.

Upon collection of the clinical data set for the first cycle, blood test showed no morbidity related to the treatment and a noticeable improvement in bone density. Glycemic values taken during each visit all remained well within reference value (60–110 mg/dl), where the patients’ average was 80 mg/dl and the range was between 75 and 86 mg/dl. The starting bone density measurement was taken on the 26th of May 2010 and a second one was taken at the end of first cycle on the 15th of February 2011. This measurement showed a BMD of 0.333 and 0.451, respectively.

**Figure 1:**
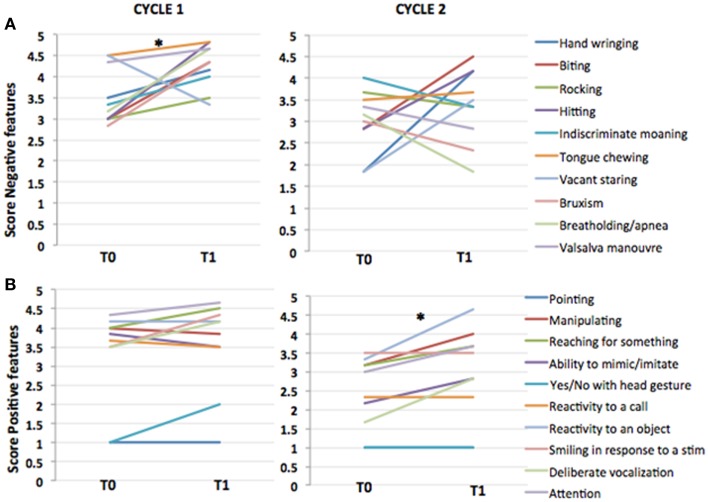
**IGF1 treatment affects different features in different cycles**. Comparison of the scores of individual features at T0 and T1 for negative **(A)** and positive **(B)** features in cycle 1 (left) and cycle 2 (right). Paired comparison with Wilcoxon test shows that IGF1 treatment improved significantly negative features in cycle 1 (Wilcoxon test, *p*-value = 0.014), but not in cycle 2 (Wilcoxon test, *p*-value = 0.07). Viceversa, for positive features the effect was significant in cycle 2 (Wilcoxon test, *p*-value = 0.016), but not in cycle 1 (0.3).

**Figure 2:**
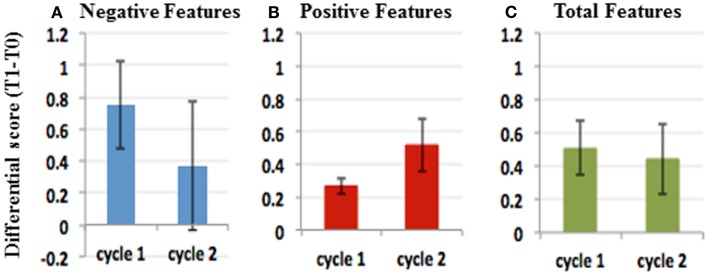
**Effects of IGF1 treatment differ in different treatment cycles**. Plots of average (T1–T0) and SE in cycle 1 and cycle 2 for Negative features **(A)**, Positive features **(B)**, and Negative plus positive features **(C)**. The data suggest that the negative features improved more in the first cycle than in the second **(A)**. Conversely, the positive features improved more in the second cycle than the first. **(B)** Considering both positive and negative features, the effect of the treatment was comparable in both cycles **(C)**.

**Figure 3:**
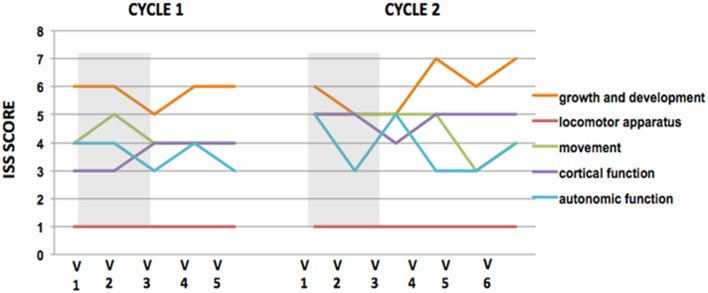
**ISS scores plots during IGF1 cycles of treatment**. ISS scores reveal that IGF1 improves autonomic function. Plots of International Severity Scale (ISS) score at each visit during cycle 1 (left) and cycle 2 (right) of IGF1 administration. Gray rectangles show the IGF1 Administration. Higher scores represent worst performance. In both cycles, the IGF1 treatment improved the autonomic function, while the other parameters remain unchanged or worsened between the beginning and the ending of each cycle. Note that the improvement is not maintained between cycles.

Analysis of cognitive and social abilities show mixed results in positive and negative RTT features, in terms of improvement and disease progression (Figures 1 and 2). Figures 1 and 2, representing video analysis, note that the negative features (9 out of 10) increased more than positive features (5 out of 10). Hitting and breath-holding/apnea were the most improved negative features. Smiling in response to a stimulation, deliberate vocalization, and yes/no with head gesture were the most improved positive features. The patient shows an improvement in height percentile during the course of IGF1 treatment in almost immediate results, however, it returned to previous percentile among the discontinuation of the IGF1 treatment. No other significant correlations to the IGF1 treatment can be made from Table 1. In the ISS evaluation, we noted a starting score of 4 in autonomic function, at the first dose of the cycle; and upon the last dose, the patient reduced to a score of 3 in autonomic function. This implies that the autonomic function restored as a result of IGF1 treatment. Another improvement noted by the ISS score was in growth and development from an ISS score of 6, at first treatment, to a score of 5, at last dosage. This also implies that IGF1 treatment improved the patient’s growth and development. ISS scores are shown in Figure 3.

**Table 1:** **Growth parameters during first cycle of treatment**.

Visit	Weight (kg)	Percentile	Height (cm)	Percentile	Head circumference	Percentile	BMI
1	14.2	4	104.5	4	48	2	13
2	15.2	4	108	10	48	2	13.03
3	17	4	108.5	10	48	2	14.5
4	17	4	108.5	10	48.4	2	14.5
5	16.7	4	110.6	4	48.4	2	13.8

*This table compares the growth parameters of the RTT patient in the first cycle with the average child of same age. The patient remains in low percentiles, despite improvements in height during IGF1 application*.

### Summary of IGF1 effects after the first cycle of treatment

We found that IGF1 was well tolerated during the first cycle of treatment and did not show any side affect. The treatment produced an increase in height and BMI of the patient mostly during the first 3 months of treatment, and returned to baseline after the discontinuation of the drug (Tables 1 and 4). Significant benefits were noted as reduction of negative stereotypes, while the social and cognitive abilities did not show any significant improvement (Figures 1 and 2; Table 5). IGF1 did not show any significant effect on ISS, with the exception of the autonomic function, that appear improved also after 6 months from the last IGF1 administration (Figure 3; Table 6).

### Second cycle of IGF1 treatment

The next cycle of IGF1 treatment started on the 20th of November 2012, when the patient was 8 years and 2 months. Since the last visit, the patient is continuing the anti-epileptic therapy: Valproate (250 + 200 mg/day) and Levetiracetam (300 + 300 mg/day). The clinical examination shows that health is fair and reported: patient has slight ataxic ambulation, which required minimum support; slight hypotonia was noticeable and Babinski positive ROT hyper-excitability; wide-spread bouts of tremor often associated with tachypnea; and possible grasping of objects upon request, but released after a few seconds. The patient has expressive language that is characterized by production of varied and modulated vocalizations. The mood of the patient is serene and there is a discreet interest in the environment and in surrounding people.

The second visit of the second cycle took place on the 29th of January 2013. The patient continues to have seizures three to four times a week characterized by eyes turned in backwards. The patient has an increased in dosage of Levetiracetam (350 + 350 mg/day) and maintained Valproate (250 + 200 mg/day). The parents reported an increase in bruxism with a decrease in stereotypic mannerisms. The patient showed longer attention span, improved eye contact, and smiling. The dietary intake was good and constipation improved. The clinical examination reported: patient has slight ataxic ambulation, which required minimum support; slight hypotonia was noticeable and Babinski positive ROT hyper-excitability; wide-spread bouts of tremor often associated with tachypnea; and possible grasping of objects upon request, but released after a few seconds. The patient had expressive language characterized by production of varied and modulated vocalizations. The mood of the patient was serene and there was a discreet interest in the environment and in surrounding people. We took extra note in the slight improvement of attention: specifically indicating that the patient holds her gaze for the duration of the smile and she seems more interested in the environment. Motor stereotypes decreased slightly while bruxism increased.

The third visit took place on the 3rd of December 2013. The grandfather informs about an improvement in attention span to external stimuli throughout the visual field laterally. The communication capability is improved and characterized by the improved ability to stare at an object that the patient wants to play with or a place that the patient wants to go. He also noticed an improved grip on objects. The mother reports that the patient eats less and tremors and bruxism have increased. She also states that sleep patterns are altered by presence of numerous episodes of pavor during sleep and wake states. The clinical examination reported: patient has slight ataxic ambulation, which required minimum support; slight hypotonia was noticeable and Babinski positive ROT hyper-excitability; wide-spread bouts of tremor often associated with tachypnea; and possible grasping of objects upon request, but released after a few seconds. The patient has expressive language that is characterized by production of varied and modulated vocalizations. The mood of the patient is serene and there is a discreet interest in the environment and in surrounding people. The clinician took another extra note in the slight improvement of attention: specifically indicating, again, that the patient holds her gaze for the duration of the smile and she seems more interested in the environment. Motor stereotypes decreased slightly while bruxism increased.

Suspension of the second cycle of IGF1 occurred on the 28th of February 2013.

The following clinical observation was recorded on the 21st of May 2013. An anamnestic incident was recorded with a slightly increased frequency of seizures and every crisis has a longer duration of action. There is a constant intense tremor present. Parents observe the patient suffering from pharyngitis and a decrease in appetite. The clinical observation includes the patient being pale and sleepy, while being poorly responsive to environmental stimulation. They informed about two critical episodes in the morning with semiology and in few seconds durations. The gait was possible with minimal support and appeared ataxic. Slight hypotonia was noticeable and Babinski positive ROT hyper-excitability. Wide-spread bouts of tremor were noted in association with tachypnea. There was grasping of objects on presentation, but released after a few seconds. The stereotypes were unchanged.

Clinical data sets provided no insight into the second cycle of IGF1 treatment in blood work or growth parameters (Table 2). Glycemic values taken during each visit all remained well within reference value (60–110 mg/dl), where the patients’ average was 78.2 mg/dl and the range was between 73 and 84 mg/dl. The starting bone density measurement was taken on the 20th of November 2012 and a second one was taken at the end of the second cycle on the 21st of May 2013. This measurement showed a BMD of 0.426 and 0.465, respectively.

**Table 2:** **Growth parameters during second cycle of treatment**.

Visit	Weight (kg)	Percentile	Height (cm)	Percentile	Head circumference	Percentile	BMI
1 and 2	15	3	115	4	48	2	11.3
3	16	3	118.5	5	48.2	2	11.5
4	16.1	3	118.5	4	48.2	2	11.5
5	15.4	2	119.4	3	48.2	2	10.8

*This table shows the growth parameters during the second cycle of IGF1 treatment. There are no significant contributions to these characteristics given by the treatment. The patient maintains in low percentiles during the second IGF1 treatment cycle*.

The patient shows mixed results for positive and negative phenotypes based on the video analysis (Figures 1 and 2), however no adverse effects are noted in association with the second cycle of IGF1 treatment. Figures 1 and 2, representing video analysis, note that the negative features (4 out of 10) increased less than positive features (6 out of 10). Hand wringing and biting showed most improvement in negative features. Reactivity to an object and deliberate vocalization showed the most improvement in positive features. In the ISS evaluation: we noted a starting score of 6 in autonomic function, at the first dose of the cycle; and upon the last dose, the patient reduced to a score of 5 in autonomic function. This implies that the autonomic function restored, again, as a result of IGF1 treatment. Another consistent improvement noted by the ISS score was in growth and development from an ISS score of 6, at first treatment, to a score of 5, at last dosage. This also implies that IGF1 treatment improved the patient’s growth and development. ISS scores are shown in Figure 3.

### Summary of IGF1 effects after the second cycle of treatment

In the second cycle of treatment, there were no significant effects of IGF1 on the growth parameters of the patient (Tables 1 and 4). The scoring of the cognitive and social abilities, as well as the negative features of Rett, shows a general worsening of the conditions between the end of the first cycle and the starting of the second cycle of treatment. The second treatment with IGF1 produces a significant improvement in the scoring for the social and cognitive abilities, but no effects are visible on the negative symptoms of RTT (Figures 1 and 2; Table 6). The ISS components show a mixed effect of IGF1 on different features, with overall a long term improvement of movement and autonomic functions (Figure 3; Table 5).

### Follow-up examinations post cycles

On September 24, 2013, the patient was 9 years old with a body mass index of 10.13 (normal values between 19 and 25 kg/m^2^). The occipital–frontal cortex circumference was 48.2 cm and her weight was 14.6 kg. The patient’s results in this report came from the evaluations of the autonomic nervous system in quiet conditions. Table 3 depicts the cardiovascular examination that took place on this date.

**Table 3:** **Cardiovascular reflexes range during cycles**.

Cardiovascular reflexes	Examined values	Normal values
Cardiac vagal tone	5.63	6–19
Heart rate	90.1 beats/min	60–140 beats/min
Respiratory rate	20 breaths/min	18–28 breaths/min

*This table depicts that the patient falls with in the normal reference range for three out of the four cardiovascular reflexes that we observed. Cardiac vagal tone is just slightly under the reference value*.

**Table 4:** **Differential scores of growth parameters during cycles**.

Growth parameters	Cycle 1		Cycle 2	
Differential score	Effect	Preserve	Effect	Preserve
Weight	2.8	−0.3	1	−0.6
Height	4	2.1	3.5	0.9
Head circumference	0	0.4	0.2	0
BMI	1.5	−0.7	0.2	−0.7

*This table summarizes the differential scores for each component of the growth (listed on the left), considering the difference at the end versus the beginning of the treatment (effect) and the difference after 6 months from the last administration versus the scores on the day of the last administration (preserve). The original scores are those reported in Tables 1 and 2. The improvements are highlighted in green, and the worsenings are highlighted in red*.

The results showed normal breathing patterns for 21.2–35.9% of mixed tachypnea breath, 12.2% of deep breaths, 7.4% of breath-holding episodes, 8% of hyperpnea, and 3.8% of apnea episodes. The average heart rate recorded is about 90 beats per minute, while the mean values of CVT in relaxation amounted in about 5.6; where the registered CVT peaks results are of average and significant.

The phenotype of the cardiopulmonary patient was classified as forced (40.2%). The general autonomic framework was that of a basal level vagal tone, which is slightly lower than normal. There were multiple types of respiratory rhythm abnormalities that were indicative of instability within the respiratory centers of the brainstem.

On 11 March 2014, a second follow-up examination was recorded. The parents noted some changes in seizure related events. Since November of 2013, the patient took Carbamazepine (25 mg in the morning and 50 mg in the evening), which was suspended while being treated with Levetiracetam. The latest crisis occurred a week before this examination where the patient had a cluster of six episodes on the same day. The patient grew agitated and had difficulty falling asleep, so Valium was given by the parents.

At this time, the patient was currently attending third grade class with support and rehabilitative physiotherapy continues 2 days a week (partially at ASL and partially privately) with swimming integrated. She was on the following AEDs: depakote (250 mg in morning and 250 mg in the evening), Carbamazepine (5 mg in morning and 50 mg in the evening), and Pepidax.

The general health conditions were satisfactory. The patient was able to autonomously maintain the sitting position with ataxia of the trunk and ambulation requires support. Tremors were noticed and the detection of bruxism. Scoliosis was absent, while there was reduced muscle tropism. The patient made little facial expression, with eye contact being only possible if the child is evoked several times; language was absent. During the visit, there were no manual stereotypes; however, the parents report it occurred when the patient was tired. The patient showed fluctuation in vigilance, and during the visit the child was alert and responsive. During neurological examinations, the child had suddenly fallen asleep for few minutes at a couple of occasions. The physician recommended that it was advisable for the patient to continue the rehabilitation treatment that was already in progress.

### Comparison of IGF1 effects during and after the two cycles

**Figure 4:**
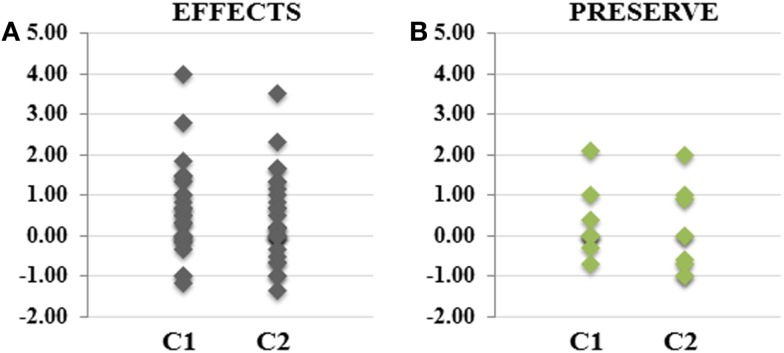
**The effects of IGF1 treatment for each cycle are comparable**. The plots represent the differential scores of growth parameters, ISS scores and Social and cognitive scores, before and after IGF1 Administration [EFFECTS **(A)**], and in follow-up studies [PRESERVE **(B)**]. Although we observe a slightly major efficacy of IGF1 during the first cycle of treatment, the differences are limited and they are not significant.

In order to compare the effects of IGF1 between the two cycles, we considered the relative values of each parameter (growth parameters, ISS scores, cognitive, and social abilities scores) during and after the termination of the drug administration, grouping together the effects during IGF1 administration and after the suspension of the drug (Figure 4). In particular, for the effects during the two cycles, was considered the differences between the last visit during treatment and the visit before treatment (Figure 4A). For the preservation of the effects after the cycles, we considered the difference between the values at the last visit post treatment and the values of the last visit during treatment (Figure 4B). We observed that the effects of the drug were comparable between the two cycles, and even with a slight positive effect during the first cycle, there were no significant differences (two tailed *t*-test, *p*-value = 0.28).

### Epileptic history during observation

The patient was enlisted into the study in March 2010, but because of an onset of epilepsy during this month the start treatment was postponed to May. There were two critical incidents between the months of March to May and on average the crisis occurred at a frequency of four/month. The patient then commenced the first cycle of IGF1 treatment on the 25th of May 2010. The epileptic treatment during this time was Valproate. From the time of the previous visit to the next visit on the 31st of August 2010, there were five critical episodes reported while on the Valproate therapy, on average the crisis during IGF1 administration have the same intensity and frequency than before the treatment. More frequent critical incidents occurred between the second and third visit, where the patient was on Valproate. Following this visit, there was the suspension of IGF1 on the 27th of November in 2010. On the fourth visit, 15th of February 2011, after the suspension of IGF1 administration, the crisis appeared in clusters on several occasions. At this point, the epileptic therapy was adjusted to Valproate plus Frisium (for when the patient has many crises). By the fifth visit on the 24th of May 2011, critical episodes became more frequents and occurred on almost a daily basis. The therapy was Valproate, while Frisium was suspended. Visit six, on the 21st of October 2011, indicated critical episodes occurring upon awakening. Still the patient remained on Valproate.

The second trial start date occurred on the 20th of November of 2012. Critical episodes are daily and the epileptic therapy was Valproate and Levatiracetam. The second visit showed similar critical episodes on a daily basis. Suspension of IGF1 occurred on the 28th of February 2013. The third visit took place on the 12th of March 2013, where the critical episodes remain daily. The crisis did not change with the administration of IGF1 treatment and the epileptic therapy remained on Valproate and Levatiracetam. On the 24th of September 2013, the critical episodes were reported the same on a daily basis. During this fourth visit, the patient was on the same epileptic treatment. On the 28th of October 2013, the epileptic therapy was changed: Levitiracetam was removed and Carbamazepine was added. The sixth visit, on the 3rd of November 2014, reported that Carbamazepine caused a thinning out of critical episodes, but they were clustered. The epileptic therapy was carbamazepine and valproic acid.

## Discussion

The use of IGF1 represents an appealing therapy for RTT. Due to IGF1 affecting multiple symptoms of RTT in animal models, its noticeable benefits from pediatric use, and its physical properties allowing it to cross the blood brain barrier; pilot trials (12, 13), indicated that IGF1 is safe and tolerable for treatment of RTT patients.

Considering the finding that any treatment for RTT should be administered for life (8), our research addresses the issue of multiple IGF1 treatments and tolerability in children with RTT syndrome. During our first study (12), we observed the major improvements during the first weeks of IGF1 treatment, and for this reason, we decided to reduce the time of the second treatment to 20 weeks. These findings are in line with the study by Khwaja et al. (13), where the amount of IGF1 in the serum decreases during the administration period, and the authors suggest that an adjustment of the dosage and administration time could be more beneficial to the Rett patients.

Although our study is mostly oriented in confirming the safety and tolerability of IGF1 through multiple cycles, we observed an overall improvement of autonomic function during IGF1 treatment, and then a lessening of improvement upon suspension of IGF1 (Figure 3; Table 5). We recorded no hypoglycemia, which indicates that IGF1 shows good toleration in patients. This is important because it is one of the main risks associated with IGF1 treatment (13).

**Table 5:** **Differential scores of social and cognitive abilities during cycles**.

(T1–T0) score	Negative features		(T1–T0) score	Positive features	
	Cycle 1	Cycle 2		Cycle 1	Cycle 2
Hand wringing	0.67	2.33	Pointing	0.00	0.00
Biting	1.33	1.67	Manipulating	−0.17	0.83
Rocking	0.50	−0.33	Reaching for something	0.50	0.50
Hitting	1.83	1.33	Ability to mimic/imitate	−0.33	0.67
Indiscriminate moaning	0.67	−0.67	Yes/No with head gesture	1.00	0.00
Tongue chewing	0.33	0.17	Reactivity to a call	−0.17	0.00
Vacant staring	−1.17	1.67	Reactivity to an object	0.00	1.33
Bruxism	1.50	−0.67	Smiling in response to a Stim	0.83	0.00
Breath-holding/apnea	1.50	−1.33	Deliberate vocalization	0.67	1.17
Valsalva maneuver	0.33	−0.50	Attention	0.33	0.67
Overall	7.83	3.67	Overall	3.17	5.17

*This table summarizes the differential scores for negative and positive features of the social and cognitive abilities assessment. Each number represents the difference of the score for the specific ability at the end of the treatment (T1) versus the beginning of the treatment (T0). Improvements are therefore positive numbers and they are highlighted in green, while worsening are negative numbers and are highlighted in red*.

**Table 6:** **Differential scores of ISS scores during cycles**.

ISS components	Cycle 1		Cycle 2	
Differential score	Effect	Preserve	Effect	Preserve
Growth and development	−1	1	−1	2
Locomotor apparatus	0	0	0	0
Movement	0	0	0	−1
Cortical function	1	0	−1	1
Autonomic function	−1	0	0	−1

*This table summarizes the differential scores for each component of the ISS (listed on the left), considering the difference at the end versus the beginning of the treatment (effect) and the difference after 6 months from the last administration versus the scores on the day of the last administration (preserve). The improvements are highlighted in green, and the worsenings are highlighted in red. Note that the decrease in the scoring are improvements for the ISS; on the contrary, increases in the scoring represent worsening*.

In this study, similarly to what reported in the past and by other researchers (12, 13), we did not observed any specific influence of IGF1 treatment on epileptic episodes: the appearance of the epileptic crisis preceded the first cycle of IGF1 administration, and the intensity and frequency of the episodes remained on average the same before and after the treatment. The intense episodes reported between the second and third week during the first cycle of treatment, can be due to an endogeneous evolution of the crisis, as suggested also by the increased frequency after the interruption of the treatment (which required an adjustment of the therapy). Before starting the second IGF1 administration, the crisis had a daily frequency and they remained unaltered during the whole cycle of treatment. These observations are in accordance to the literature in pediatric syndromes, where IGF1 is not associated to an increase in seizures, but rather epileptic phenotype is associated to decreased IGF1 levels in the serum (14, 15). On the contrary, it is possible that the seizure medication hindered the positive effects of IGF1. It would be interesting to compare the effects of IGF1 administration in patients treated with valproate versus patients treated with alternative AEDs.

Insulin-like growth factor 1 has also been considered to be more effective when given in acute intermittent pulses, versus chronic dosing. One noted side effect from IGF1, was that long term IGF1 administration has linkage with potential puberty effects; which is already accelerated in RTT (13). Being that IGF1 has a strong feedback system, patient tolerance over cycles is worth exploring into for future studies. This implies the evaluation of lower doses in life-long treatment. An assessment of total effects on single analyzed relative parameters (ISS scores, cognitive and social abilities, and growth parameters) showed that the effects observed with the first cycle of treatment were not significantly different from those in the second cycle of treatment (Figure 4, two tailed *t*-test *p*-value = 0.28).

While there were some phenotypic benefits shown by the assessment of social and cognitive abilities, nine of the improvements remained after the first cycle was over, and no phenotypic improvement remained in the second trial as different phenotypes improved aside from attention. However, we observed motor control as another form of improvement. However, our research insight into motor control improvement may be a result of subjectivity further research is suggested to elucidate the validity of this potential benefit. Our analysis confirms the results of Zoghbi’s team, that whatever treatment is decided for RTT syndrome patients, it must be for life (8).

The two studies of IGF1 safety and tolerability in RTT patients (12, 13), showed similar results: IGF1 did not produce hypoglycemia and it is not associated to seizures, and overall is well tolerated by patients. In addition, we observed an improvement in autonomic function, and Khwaja and colleagues concluded that motor ability showed no measurable improvement, but also found that respiratory complications associated with RTT syndrome showed improvement (13).

One factor to consider in our findings is the environmental surrounding that the patient experienced during and after the IGF1 treatment. The patient lived with the mother during the time of treatment. The mother suffers from anxiety and seizures, which may have limited the benefits of IGF1 treatment due to high stress levels being perceived by the patient. The consideration is due to a follow-up communication, post IGF1 second cycle treatment, where the grandparents noted an immediate amelioration of the patient’s phenotypic mannerisms. The stressful environment could have caused misperception of the severity level to phenotypic hindrances, which are RTT associated.

The personal environmental surrounding, together with the onset and progression of the epileptic crisis are two of the limits of this case study for assessing the benefits of IGF1 therapy. Appropriate double blind and placebo controlled studies are necessary to define the efficacy of IGF1 treatment. However, considering the rarity of RTT (1:10,000), and the recent discovery of IGF1 as possible Rett treatment, data coming from a single case study are nonetheless useful to further develop the treatment.

Our research show that repeated treatments with IGF1 is safe and tolerable for the patient. This finding along with other findings on IGF1 treatment with RTT, should encourage further research in IGF1 treatment for additional neurodevelopmental disorders.

## Concluding Remarks

Our research shows that repeated treatments with IGF1 are safe and tolerable for the patient. We observed improved autonomic function and social and cognitive abilities in each cycle but the benefits were lost between cycles. The results in this clinical case study suggest that IGF1 is beneficial for our patient, but a larger cohort of patients and proper clinical trials are requested to establish the overall efficacy in RTT.

## Conflict of Interest Statement

The authors declare that the research was conducted in the absence of any commercial or financial relationships that could be construed as a potential conflict of interest.
